# A clinical role of staging laparoscopy in patients with radiographically defined locally advanced pancreatic ductal adenocarcinoma

**DOI:** 10.1186/s12957-016-0767-y

**Published:** 2016-01-20

**Authors:** Sohei Satoi, Hiroaki Yanagimoto, Tomohisa Yamamoto, Hideyoshi Toyokawa, Satoshi Hirooka, So Yamaki, Singh Sapam Opendro, Kentaro Inoue, Taku Michiura, Hironori Ryota, Yoichi Matsui, Masanori Kon

**Affiliations:** Department of Surgery, Kansai Medical University, 2-5-1, Shin-machi, Hirakata-City, Osaka 573-1010 Japan

**Keywords:** Staging laparoscopy, Peritoneal carcinomatosis, Occult distant organ metastasis, Second-line chemotherapy, Locally advanced PDAC

## Abstract

**Background:**

The aim of current study is to verify usefulness of staging laparoscopy (stag-lap) for patient’s selection and to find prognostic factors in patients with radiographically defined locally advanced (RD-LA) pancreatic ductal adenocarcinoma (PDAC).

**Methods:**

The LA disease was defined as an unresectable disease without distant organ metastasis based on resectability status of NCCN guideline in this study. Stag-lap was performed in 67 patients with RD-LA (2007–2012) which were divided into 4 groups according to metastatic site: group CY (peritoneal fluid or washing cytology positive and without any distant organ metastasis); group P (peritoneal dissemination); group L (liver metastasis); group LA (peritoneal fluid or washing cytology negative and without any distant organ metastasis). Clinical backgrounds, survival curves, and prognostic factors were investigated.

**Results:**

There were 16 patients in CY group (24 %), 13 patients in P group (19 %), 10 patients in L group (15 %), and 28 patients in LA group (42 %). Median survival time was 13 months in CY group and 11 months in LA group, which was significantly better than 7 months in P group, respectively (*p* < 0.05). The rate of emergence of ascites in LA was significantly better than in CY or P groups (*p* < 0.05). Multivariate analysis showed that the presence of partial response and administration of second-line chemotherapy were significantly independent prognostic factors.

**Conclusions:**

The majority of PDAC patients with RD-LA had occult distant organ metastasis. Clinical features and survival curves were different depending on the site of occult distant organ metastasis. Administration of second-line chemotherapy and responsiveness to chemotherapy were associated with favorable prognosis. Staging laparoscopy should be routinely performed in patients with RD-LA PDAC (UMIN000019936).

## Background

Pancreatic ductal adenocarcinoma (PDAC) is a lethal disease with poor prognosis, even in patients who have undergone resection with curative intent [1]. At the time of diagnosis, the majority of patients have unresectable disease with or without distant organ metastasis on imaging modality [1, 2]. Despite the advances and resolution improvement of imaging technologies, less-invasive staging modalities are still limited in their ability to identify accurately metastatic disease of small volume, resulting in inappropriate patient selection for therapy [3].

Staging laparoscopy is a minimally invasive procedure that can identify occult distant metastases, resulting in appropriate patient selection for chemotherapy or chemoradiation therapy [3]. Stefanidis et al. [4] stated in their review that staging laparoscopy has an important role in the staging algorithm of PDAC patients and will likely continue to be a valuable tool. Diagnostic laparoscopy guidelines [5] have proposed that staging laparoscopy should be considered after high-quality imaging studies have excluded metastatic disease in appropriately selected patients with locally advanced pancreatic adenocarcinoma.

We had some clinical questions on the diagnoses and clinical courses in radiographically defined locally advanced (RD-LA) patients, such as the frequency of occult distant organ metastasis, the survival difference according to the occult distant organ metastatic site, and the prognostic factors in this patient population. Therefore, since 2007, we have introduced staging laparoscopy in PDAC patients with RD-LA for further exploration of minute distant organ metastases which were not detected by conventional staging modalities [3]. In the current study, we retrospectively assessed the rate of minute distant organ metastasis in PDAC patients with RD-LA, compared the clinical background and survival rate according to metastatic site, and analyzed the prognostic factor**s**.

## Methods

A retrospective study was conducted using a prospective database. A total of 70 out of 395 patients with PDAC (267 unresectable and 128 resected) presented with RD-LA between January 2007 and December 2012 at Kansai Medical University Hospital, Osaka, Japan. Three patients refused to undergo staging laparoscopy and therefore, ultimately, staging laparoscopy was performed in 67 patients with RD-LA. The LA disease was defined as an unresectable disease without distant organ metastasis based on resectability status of NCCN guideline [6] in this study as follows; greater than 180° superior mesenteric artery encasement and any celiac abutment for pancreatic head PDAC; superior mesenteric artery or celiac encasement greater than 180° for pancreatic body and tail PDAC; unreconstructible superior mesenteric/portal vein occlusion and aortic invasion or encasement for both PDAC. These 67 patients were divided into 4 groups according to metastatic site; group CY (cytology positive and without any distant organ metastasis); group P (peritoneal dissemination); group L (liver metastasis); group LA (pure locally advanced disease, without positive cytology and any distant organ metastasis). Clinical backgrounds and survival curves according to metastatic site were compared among groups and investigated as a prognostic factor for this population. All patients were followed up for at least 1 year.

All patients had a pathological evidence of PDAC before or during staging laparoscopy. Resectability was assessed with cine-imaging MDCT, using the Aquilion® CT system (Toshiba Medical Systems, Tochigi, Japan). Arterial- and portal-phase images were collected using a 1.0 × 64 mm detector configuration. After reconstruction of the raw scans, data from serial 1.0-mm-thick slices with a 0.5-mm interval were transferred to a workstation (AquariusNet Viewer, TeraRecon Inc., San Mateo, CA, USA). After creating two- and three-dimensional coronal and sagittal anatomical reconstructions, the cine images were evaluated by an experienced hepatopancreatobiliary surgeon and a consultant radiologist. The clinical response rate was based on the Response Evaluation Criteria in Solid Tumors (RECIST) [7], and partial response was defined as at least a 30 % decrease in the sum of diameters of target lesions, taking as reference the baseline sum diameters.

Patients with resectable, borderline resectable, and metastatic PDAC based on cine-imaging MDCT were excluded from the study. Patients with cancer of the pancreatic body and tail, with celiac trunk invasion, but with no invasion of the superior mesenteric and pancreaticoduodenal arteries, were also classified as potential candidates for resection with curative intent in this institution and thus excluded from participation. Positron emission tomography (PET) scanning was performed in patients with suspicion of metastatic disease in the lung, cervical, or para-aortic lymph nodes on cine-imaging MDCT. All patients with a suspicion of distant organ metastasis diagnosed by PET scanning were excluded after confirmation of adenocarcinoma in the biopsy specimens from locations such as the liver, lung, cervical lymph nodes, and bone. Although the patients with omental cake or multiple peritoneal nodules greater than 10-mm diameter were also excluded from the study, patients with suspicion of peritoneal metastasis less than 10-mm diameter on MDCT or ascites limited to the Pouch of Douglas were recruited.

The location of the metastatic site diagnosed by the staging laparoscopy was recorded. Patients with occult distant organ metastasis were treated with gemcitabine or S-1-based chemotherapy or combination chemotherapy of gemcitabine and S-1. Radiation therapy was added as the first-line treatment concomitant with chemotherapy in 15/28 patients with locally advanced tumor or 5/16 patients with positive cytology. Second-line chemotherapy (gemcitabine, S-1, or gemcitabine and S-1) was administered to patients with their consent and if they also had a good performance status. The time of development of ascites according to the site of distant organ metastasis was also recorded.

Informed consent was obtained from each patient included in the study, in accordance with the provisions of the Declaration of Helsinki. Patient data were obtained from the prospective database of pancreatic disease at Kansai Medical University Hospital.

### Staging laparoscopy

Briefly, after establishing the pneumoperitoneum through a 12-mm trocar inserted at the umbilical area, a flexible laparoscope was inserted and two additional 5-mm ports were placed. First, an inspection focusing on the presence or absence of nodules on the parietal peritoneum and the liver surface was carefully performed. Second, a cytological examination was performed in cases where ascites was present. When there was no ascitic fluid, the peritoneal cavity was washed with 100 ml of physiologic saline solution. After placing the patient in Trendelenburg’s position, the presence or absence of nodules in the Pouch of Douglas was checked by inserting the laparoscope through filled water into the Pouch of Douglas. Subsequently, cytologic washings from the Pouch of Douglas were obtained for pathological examination. Third, the entire mesentery was examined by grasping the small intestine from the first jejunal loop to the ileocecal junction to locate the minute peritoneal nodules. Biopsies were taken from any regions suspected of containing metastatic nodules. Neither the lesser nor the greater sac was opened in this study. This clinical study was registered as UMIN000019936.

### Statistical analysis

Data are expressed as median values and ranges. Clinical background and clinical course were compared according to metastatic site in Table 1. Continuous or categorical variables were compared by Mann–Whitney *U* test, chi-square test, or Fisher’s exact test as appropriate. Overall survival curve, defined as the time from initial treatments to death or the last follow-up date, were compared using the log-rank test according to metastatic site. In addition, profound factors identified by the univariate analysis were further examined by multivariate Cox proportional-hazard models to determine independent significant factors for survival. The hazard ratio and 95 % confidence intervals were calculated for all estimates. A two-tailed *p* value less than 0.05 was considered to be statistically significant. Statistical analyses were performed using SPSS version 18.0 for Windows (Chicago, IL, USA).

**Table 1:** Patient characteristics and clinical course in each group

Parameters	LA (*n* = 28)	CY (*n* = 16)	P (*n* = 13)	L (*n* = 10)
Gender (M/F)	14:14	11:5	6:7	6:4
Age (years)	66 (47–82)	63 (41–85)	69 (42–78)	63 (55–75)
PS 0/1 vs 2	26:2	16:0	12:1	8:2
Location (Pbt/Ph)	11:17 (39)	6:10 (38)	10:3 (77)	6:4 (60)
Tumor size (mm)	41 (23–76)	41 (27–91)	47 (21–88)	37 (28–85)
Size > 39.5 vs low	15:13	9:7	9:4	3:7
DM (yes/no)	5:23	3:13	5:8	4:6
Obstructive jaundice (yes/no)	15:13	7:9	4:9	5:5
Bilirubin (mg/dl)	0.8 (0.3–10)	0.7 (0.4–2.8)	0.6 (0.4–1.3)	0.9 (0.5–1.8)
Albumin (g/l)	3.6 (2.3–4.5)	3.8 (2.9–4.6)	4.0 (2.7–4.6)	4.0 (3.3–4.7)
Hemoglobin (g/dl)	11.7 (8–14.7)	11.9 (10.5–14.9)	12.4 (10.2–14.8)	13.3 (11–14.9)
CA19-9 (IU/l)	232 (1–3978)	371 (18–13,400)	385 (6–18,977)	997 (66–34,408)
CTx vs BSC	23:5	16:0	9:4	9:1
Duration of CTx (months)	7.5 (0–64)	10 (1.5–53)	3 (0–10)	6 (0–17)
GS (yes/no)	12:16	7:9	2:11	3:7
Radiation (yes/no)	15:13	5:11	0:13	0:10
Second-line chemotherapy (yes/no)	18:10	10:6	1:12	7:3
CR/PR vs SD/PD	9:19	6:10	2:11	2:8
1-year survival rate	50 %	63 %	31 %	20 %
Adjuvant surgery	1/28	2/16	0	0
Development of ascites within 1 year	10/28 (36 %)	7/16 (44 %)	11/13 (85 %)	5/10 (50 %)

Data are expressed as the median (range) or *n* (%)

*LA* locally advanced, *CY* positive cytology, *P* peritoneal metastasis, *L* liver metastasis, *M* male, *F* female, *PS* performance status, *Ph* pancreas head, *Pbt* pancreas body and tail, *DM* diabetes mellitus, *CA19-9* carbohydrate antigen 19-9, *CTx* chemotherapy, *BSC* best supportive care, *GS* gemcitabine and S-1, *CR* complete response, *PR* partial response, *SD* stable disease, *PD* progressive disease

## Results

### Outcome measures of staging laparoscopy

Median operative time was 67 min (34–168) and median extent of blood loss was 0 ml (0–168). Past history of previous surgery was found in 12 out of 67 patients (18 %). Three patients needed open mini-laparotomy converted from staging laparoscopy because of severe adhesion due to previous surgeries. Biopsy specimens were obtained from 43 patients (64 %) for evaluating if occult distant organ metastasis was present. One patient incidentally had intestinal perforation during staging laparoscopy, and primary closure was immediately done. Her postoperative course was uneventful and underwent chemotherapy without delay.

Exploration using staging laparoscopy clearly demonstrated the presence of occult distant organ metastasis in 39 out of 67 patients (58 %). There was positive cytology in 16 patients (CY, 24 %), peritoneal dissemination in 13 patients (P, 19 %), liver metastasis only or concomitant with peritoneal metastasis in 10 patients (L including 3 patients with concomitant peritoneal metastasis, 15 %), and locally advanced disease in 28 patients (LA, 42 %), respectively.

### Comparison of clinical backgrounds and clinical course according to metastatic site

**Fig. 1:**
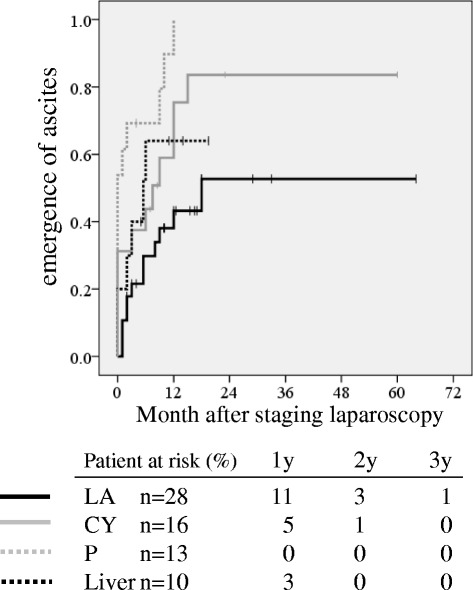
The rate of emergence of ascites according to metastatic site diagnosed by staging laparoscopy. Figure 1 shows the curve of emergence of ascites in patients with locally advanced PDAC (LA, *solid black line*), with positive cytology (CY, *solid grey line*), with peritoneal metastasis (P, *dotted grey line*), and with liver metastasis (L, *dotted black line*). There were significant differences in the rate of emergence of ascites between LA and P (*p* < 0.001) and LA and CY (*p* = 0.033) groups

With regard to the clinical backgrounds and the clinical course, as shown in Table 1, the L or P groups had a higher frequency of pancreatic body and tail cancer, compared with the LA or CY groups. Addition of radiation therapy during chemotherapy was frequently performed in the LA and CY groups according to the departmental policy. The clinical response rate, as based on the RECIST [7], was higher in the LA or CY groups than in either the P or L groups. When the rate of emergence of ascites according to the site of occult distant organ metastasis was compared, it was significantly lower in the LA group than in CY or P group (Fig. 1, *p* < 0.05). Development of ascites within 1 year after initial treatment was found most frequently in patients in the P group (11 out of 13 patients, 85 %), which was higher than in the other 3 groups. Second-line chemotherapy was administered less frequently in P group (1 out of 13 patients, 8 %), relative to the other three groups (Table 1). Moreover, duration of chemotherapy in P group was shorter than in the other groups.

**Fig. 2:**
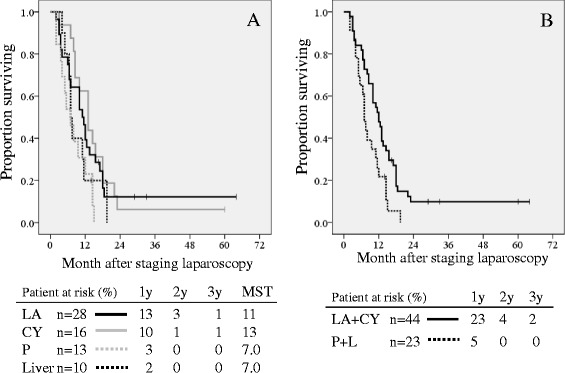
Survival curves according to metastatic site diagnosed by staging laparoscopy. **a** shows survival curve of patients with locally advanced PDAC (LA, *solid black line*), with positive cytology (CY, *solid grey line*), with peritoneal metastasis (P, *dotted grey line*), and with liver metastasis (L, *dotted black line*). There were significant differences between the survival curves of P and CY (*p* = 0.007) and P and LA (*p* = 0.04) groups. **b** shows survival curves of patients with locally advanced PDAC and positive cytology (LA + CY, *solid black line*) and with peritoneal metastasis and liver metastasis (P + L, *dotted black line*). The survival curve in LA + CY groups was significantly better than in P + L groups (*p* = 0.011)

Median survival time was 13 months in the CY group (including 2 patients who underwent surgical resection), 11 months in the LA group (including 1 patient who underwent surgical resection), and 7 months in the P and L groups, respectively. Actual 1-year survival rates in the 4 groups were as follows: 46 % (LA), 63 % (CY), 23 % (P), and 20 % (L). No patient in the P and L groups was alive beyond the 2-year follow-up. Thus, as shown in Fig. 2a, the overall survival curve in P group was significantly worse than in either the LA or CY groups (*p* = 0.04 and *p* = 0.007, respectively). No significant differences in the survival curves were seen between CY and LA, P and L, or LA and L groups. As shown in Fig. 2b, the survival curve in the LA + CY groups was significantly better than in the P + L groups (*p* = 0.011). In the LA or CY groups, additional radiation therapy was administered to approximately 30–50 % of patients, PR according to RECIST [7] was observed in approximately 35 % of patients, and second-line chemotherapy was administered to around 60 % of patients. In this study, out of 44 patients in LA and CY groups, conversion surgery was performed in two patients in the CY group (one alive after 60 months; the other died at 18 months, respectively) and one patient in the LA group (alive after 17 months). The multivariate analysis showed that the presence of partial response and administration of second-line chemotherapy were significantly independent favorable factors for prognosis (Table 2).

**Table 2:** Uni- and multivariate analyses for prognosis

Variable	Univariate analysis		Multivariate analysis			
	*p*	Hazard ratio (95 % CI)	Estimate	SE	p	Hazard ratio (95 % CI))
CR/PR vs SD/PD	<0.001	0.23 (0.12–0.45)	−1.30	0.37	<0.001	0.27 (0.13–0.56)
Second-line chemotherapy none vs done	<0.001	3.60 (2.03–6.37)	0.98	0.40	0.013	2.67 (1.23–5.78)
GS none vs done	0.009	2.05 (1.19–3.52)	0.21	0.34	0.53	1.24 (0.64–2.39)
Radiation none vs done	0.009	2.21 (1.22–4.01)	0.13	0.39	0.73	1.14 (0.53–2.45)
No ascites vs development of ascites at 1 year	<0.001	0.39 (0.23–0.65)	−0.12	0.31	0.70	0.89 (0.48–1.63)
LA/CY vs P/L	0.015	0.51 (0.30–0.88)	−0.11	0.32	0.72	0.89 (0.48–1.68)

*CI* confidential interval, *SE* standard and error, *CR* complete response, *PR* partial response, *SD* stable disease, *PD* progressive disease, *GS* gemcitabine and S-1, *LA* locally advanced, *CY* positive cytology, *P* peritoneal metastasis, *L* liver metastasis, *CTx* chemotherapy, *BSC* best supportive care

## Discussion

The majority of patients with PDAC have unresectable disease at initial presentation, and their prognosis is extremely poor. The median survival time of these patients ranges from 6 to 12 months in spite of developed, advanced, and newly provided regimens of chemotherapy [8–11]. Accurate staging is necessary for selecting patients who should be curatively resected, for introducing new regimens of chemotherapy or chemo(radio)therapy, or for conducting a clinical trial. Several articles have reported that the use of staging laparoscopy to further explore locally advanced PDAC, initially diagnosed by CT scan, has identified the presence of occult distant organ metastasis [4, 5, 12–17]. However, less information is available on clinical background and clinical course according to occult distant metastatic site diagnosed by staging laparoscopy in patients with RD-LA PDAC. Understanding the clinical features according to the site of occult distant organ metastasis is important for the development of new strategies and improving prognosis in patients with locally advanced PDAC.

We have focused on RD-LA PDAC defined by the NCCN guidelines [6], excluding resectable, borderline resectable, and metastatic diseases. The results of staging laparoscopy in this study showed occult distant organ metastasis in 58 % of patients including positive cytology in 24 %, peritoneal dissemination in 19 %, and liver metastasis in 15 %, respectively. Finally, pure locally advanced disease without minute distant organ metastasis was found in 42 % of patients only. Clark et al. [12] reported that diagnostic laparoscopy for borderline resectable and unresectable locally advanced diseases showed occult distant organ metastasis in 58 of 202 patients (29 %) including positive peritoneal lavage cytology in 20 % (*n* = 41), liver metastasis in 13 % (*n* = 26), and peritoneal metastases in 3 % (*n* = 5) (Table 3). Relative to Clark et al. [12], our study consisted of a more limited population of unresectable, locally advanced PDAC. As shown in Table 3, compared with other studies, our study consisted of high frequency of pancreatic body and tail cancer, which was associated with unresectability and the presence of occult distant organ metastasis [4, 5]. In our experience, we had an aggressive attitude towards surgical resection, even in patients with portal or superior mesenteric vein invasion, common hepatic artery invasion, and celiac axis invasion for pancreatic body cancer. The definition of local unresectability for our patients was strictly limited. Moreover, we also recruited patients with suspicion of peritoneal metastasis less than 10-mm diameter on MDCT or ascites limited to the Pouch of Douglas. Therefore, this could explain why a high incidence of occult distant organ metastasis (58 %) and peritoneal metastasis (19 %) was found in our study.

**Table 3:** Review of the recently published articles on staging laparoscopy in patients with locally advanced pancreatic cancer

Authors	Year	Duration	Number of patients	Rate of patients	Laparoscopic findings (%)						MST (months)
					True LA	Occult met	CY	P	Liver	P/Liver	
Clark et al. [12]	2010	00–08	202	22 %	144 (71 %)	58 (29)	41 (20)	5 (3)	26 (13)	-	13
Morak et al. [13]	2009	95–07	68	-	44 (65)	24 (35)	14 (21)		5 (7)	2 (3)	11.7
Contreras et al. [14]	2009	02–06	33	15 %	22 (67)	11 (33)	-	7 (21)	4 (12)		
Shoup et al. [15]	2004	94–00	100	31 %	63 (63)	37 (37)	12 (12)		18 (18)	7 (7)	-
Current study	-	07–12	67	51 %	28 (42)	39 (58)	16 (24)	13 (19)	7 (10)	3 (4)	10

*Pts* patients, *LA* locally advanced, *met* metastasis, *CY* positive cytology, *P* peritoneal metastasis, *MST* median survival time

We have introduced palliative chemotherapy in patients with distant organ metastasis including peritoneal metastasis, and chemo(radio)therapy for tumor remission in patients with LA or CY. The clinical findings by staging laparoscopy can provide to change the paradigm of the treatment in patients with RD-LA PDAC. Actually, peritoneal and/or liver metastasis was observed in 34 % of patients, who were treated with palliative chemotherapy. Thus, the results of staging laparoscopy changed the treatment according to distant organ metastatic sites. Considering the high frequency of patients with occult distant organ metastasis, staging laparoscopy should be routinely performed in patients with RD-LA PDAC.

With regard to the comparison of survival curves according to the site of occult distant organ metastasis, P or L groups had a worse prognosis than either the CY or LA groups in our study. Actual 1-year survival rate was 46 % in LA, 63 % in CY, 23 % in P, and 20 % in L groups. In the LA or CY groups, high frequency of additional radiation, PR according to RECIST, second-line chemotherapy and conversion surgery might result in better prognosis. Appearance of partial or complete response after chemo(radio)therapy can sometimes lead to subsequent surgical resection. It has been reported that the overall survival rate in patients with initially unresectable PDAC who underwent surgical resection after favorable response to anti-cancer treatments over a period of time was similar to that in patients with resectable PDAC [18, 19]. Even in patients with initially unresectable disease, long-term survival can be expected if an unresectable tumor was surgically resected after tumor shrinkage. Advanced chemotherapy or chemoradiation therapy may have a role in downsizing an unresectable tumor sufficiently to render it resectable. Thus, a new strategy to shrink the tumor in LA or CY group should be introduced where possible to allow for subsequent surgical resection, which is associated with long-term survival.

The multivariate analysis showed that the presence of partial response and administration of second-line chemotherapy were significantly independent favorable factors for prognosis (Table 2). The curve of emergence of ascites in CY or P group was significantly worse than in LA group (Fig. 1, *p* < 0.05). The P group in particular had ascites in 85 % of patients within 1 year after initial treatment. Moreover, no patient survived longer than 2 years after initial treatment in P group. A lower frequency of second-line chemotherapy and clinical response and shorter duration of chemotherapy in P group were found in this study, probably due to emergence of ascites and poor performance status. Thus, conventional chemotherapy may not be effective for patients with peritoneal metastasis, and therefore, a new regimen to control or prevent ascites will be required for achieving better prognosis. In this regard, intraperitoneal chemotherapy may be one option for patients with peritoneal metastasis that should be investigated. A Japanese case report described the success of intraperitoneal paclitaxel combined with systemic chemotherapy of S-l and gemcitabine for locally advanced PDAC with peritoneal carcinomatosis [20]. After 20 months, all peritoneal deposits had disappeared and a radical resection of the tumor was performed. Sugarbaker et al. [21] have conducted a phase II study of hyperthermic intraoperative gemcitabine and long-term intraperitoneal gemcitabine in the adjuvant setting for resectable PDAC patients. Further protocol-based studies should be performed to investigate whether prognosis in patients with occult peritoneal metastasis can be improved or not.

The limitation of the study was the small number of patients recruited retrospectively and the presence of undetectable liver metastasis deep within the liver and/or other organ metastasis.

## Conclusions

In conclusion, staging laparoscopy is necessary for selecting patients with occult distant organ metastasis which is the majority population of patients with RD-LA PDAC. Clinical features and survival curves were different depending on the site of occult distant organ metastasis. Administration of second-line chemotherapy and responsiveness to chemotherapy were associated with favorable prognosis. Staging laparoscopy should be routinely performed in PDAC patients with RD-LA.
